# Online Learning Satisfaction During COVID-19 Pandemic Among Chinese University Students: The Serial Mediation Model

**DOI:** 10.3389/fpsyg.2021.743936

**Published:** 2021-10-05

**Authors:** Long She, Lan Ma, Anbareen Jan, Hamid Sharif Nia, Pardis Rahmatpour

**Affiliations:** ^1^Faculty of Business and Law, Taylor’s University, Subang Jaya, Malaysia; ^2^Foundation of Studies, Warwick University, Coventry, United Kingdom; ^3^Educational Development Center, Mazandaran University of Medical Sciences, Sari, Iran; ^4^Department of Nursing, Alborz University of Medical Sciences, Karaj, Iran

**Keywords:** online learning, student satisfaction, interaction, academic self-efficacy, student engagement, COVID-19 pandemic

## Abstract

The aim of this study was to investigate the relationship between interaction and online learning satisfaction, whether this relationship is mediated by academic self-efficacy and student engagement among Chinese university students during the COVID-19 pandemic. A serial mediation model was developed to examine the proposed relationship. This study employed a cross-sectional, questionnaire-based research design. A sample of 1,504 Chinese university students (*M*_age_=19.89years, *SD*_age_=1.93) from five provinces in China completed an online survey questionnaire from December 2020 to January 2021 to respond to questions on demographic characteristics and items to measure the variables in the research model. The partial least squares structural equation modeling was used to assess the measurement model and proposed serial mediation model. Data were analyzed using SmartPLS software version 3.3.2. The results of the measurement model showed good reliability and validity for all constructs. The results of the structural model and hypothesis testing showed that all hypotheses were supported in this study. Particularly, there was a significant positive relationship between interaction and online learning satisfaction (Q1), interaction and academic self-efficacy (Q2), academic self-efficacy and student engagement (Q3), and the student engagement and online learning satisfaction (Q4). In addition, the results showed that academic self-efficacy and student engagement serial mediated the relationship between interaction and online learning satisfaction (Q5). The serial mediation model explained 34.6% of the variance of online learning satisfaction. The findings shed light on the underlying mechanisms that explain students’ online learning satisfaction during the COVID-19 pandemic. Universities and policymakers need to make better decisions that ultimately could lead to students’ academic outcomes and achievement.

## Introduction

The COVID-19 declared as a pandemic by World Health Organization in 2020 has utterly disrupted educational activities, forcing most universities to a full closure, thus affecting hundreds of millions of students and educators across the globe (Shahzad et al., 2021). When traditional learning and teaching are no longer an option, online learning (synchronous or asynchronous) acts as an alternative to support the continuation of education in the midst of a pandemic with its flexibility, accessibility, and convenience (Adedoyin and Soykan, 2020; Selvanathan et al., 2020). Most higher institutions shifted from face-to-face learning to emergency remote teaching (Jan, 2020), and the motive behind such implementation was to alleviate the transmission of the coronavirus and maintain the continuation of education during the challenging times of lockdown among students and educators (Bayham and Fenichel, 2020; Wang et al., 2020).

Faculty members of universities have begun to learn and deliver online teaching to their students and are eager to understand how to produce better learning outcomes with online instructions (Shahzad et al., 2021). On the other hand, students have more control over the content and time to learn based on their individual learning needs and autonomy (Coman et al., 2020). However, this unplanned and rapid shift has raised concerns over the quality of learning, students’ academic achievement (Sahu, 2020) and satisfaction (Dziuban et al., 2015) as not much information or guidance is available on the best online teaching practices for instructors (Armstrong-Mensah et al., 2020). On the contrary, prior to the COVID-19 pandemic, online education featured with high attrition rates was downgraded as the second best preferred by students compared to traditional higher education (Muilenburg and Berge, 2005; Hassan et al., 2021).

Preliminary studies emphasize the pivotal role that student satisfaction plays in determining the success or failure of online education (Kuo et al., 2014; Rabin et al., 2019; Gopal et al., 2021) opposes the completion rates, as learners’ satisfaction reflects how they perceive their learning experiences (Kuo et al., 2014) and interprets the quality of the course instruction (Hew et al., 2020). Interaction in a fully online learning setting has been regarded as a critical factor that determines to the extent which students are satisfied with their online education (Wu et al., 2010; Cidral et al., 2018). According to Kuo et al. (2014), a high level of interaction with the instructor, other learners, or content leads to high satisfaction and thus reveals high engagement in online learning (Veletsianos, 2010). Similarly, lack of interaction often leads to poor student engagement and lower student satisfaction (Martin et al., 2018; Rahmatpour et al., 2021). It can be concluded that interaction in online learning often translates to students’ engagement in their academic activities before positively affecting students’ satisfaction (Kim and Kim, 2021).

On the other hand, academic self-efficacy has been indicated to have a positive effect on students’ engagement within the self-directed distance education nature, where students with high academic self-efficacy are more engaged in their online studies (Jung and Lee, 2018) and more likely to experience learning satisfaction (Artino, 2008). Academic self-efficacy, which is understood as students’ belief incapability to perform academically well during an online platform, has been reported to be the most predictive factor of students’ satisfaction (Shen et al., 2013; Jan, 2015). As aforementioned, prior studies indicate the significant role of interaction (Enkin and Mejías-Bikandi, 2017), academic self-efficacy (Shen et al., 2013), and students’ engagement in the online classrooms (Robinson and Hullinger, 2008) and their relationship to online learning satisfaction. There is a scarcity of studies investigating the mechanisms of interaction, self-efficacy, and engagement on students’ overall satisfaction. Hence, the extension of the existing research is needed.

This study adopts the theory of transactional distance (Moore, 1993), most often identified with distance learning programs (Benson and Samarawickrema, 2009). It helps identify the mechanism behind the relationship between interaction and satisfaction. Ekwunife-Orakwue and Teng (2014) argue that although the theory of transactional distance has been posited to explain the mechanisms in online learning education, few studies have identified the factors from this theory to predict a causal pathway for the mechanism of occurrence. Nevertheless, the theory recognizes interaction as a bridge to “a psychological and communications gap” in distance learning in promoting students’ overall satisfaction (Moore, 1993; Benson and Samarawickrema, 2009). Hence, this study goes one step further and suggests that academic self-efficacy and student engagement may explain the mechanism behind the relationship between interaction and online learning satisfaction among online learners, particularly Chinese online learners.

China was the first country to respond to this transition by instructing a quarter of billion full-time students to resume their studies online (OECD, 2020; World Economic World Economic Forum, 2020). Chinese online education advocates “interactivity” in online learning provides some perspectives to access online learning in our study. As students’ satisfaction reflects the effectiveness of e-Learning quality (Alqurashi, 2019), it has become very important to understand how interactions impact the e-Learning quality, especially during the pandemic when the education around the world has moved to online teaching & learning (Kumar et al., 2021). However, the literature is not exhaustive on student satisfaction in an online environment during the pandemic. It is particularly scarce in the context of developing countries, as in the case with China. Thus, the current study offers some new insights on distance learning by investigating the mechanism behind the relationship between online interaction and learners’ satisfaction from students’ perspectives with the lens of the theory of transactional distance in developing countries. Secondly, there is a regrettable paucity of research to address the serial mediation of academic self-efficacy and student engagement in the correlation between students’ satisfaction and interactions. And it is worth noting that student satisfaction is closely tied to their academic performance or achievement and also acts as an indicator to measure the success of online courses (Alqurashi, 2019). Thus, to understand student satisfaction and its relationship to interaction through student engagement, academic self-efficacy will largely assist students in achieving better online learning outcomes. On the other hand, academic self-efficacy has been supported by prior researchers on its impact on student engagement (Bong and Skaalvik, 2003) has been only measured at a task-specific level and has not yet been widely measured at a general level (Ferla et al., 2009). Thus, the study holds significance in opening up a new perspective for educators and policymakers on how to effectively plan for the implementation of distance learning in any situation in the future.

### Literature Review

#### Online Learning Satisfaction

Learning satisfaction represents learners’ feelings and attitudes toward the learning process or the perceived level of fulfilment attached to one’s desire to learn, caused by the learning experiences (Topala and Tomozii, 2014). In the online context, satisfaction has been found to be one of the most significant considerations influencing the continuity of online learning (Moore and Kearsley, 2011; Parahoo et al., 2016). Previous research on online learning has shown that learners’ satisfaction is a critical indicator of learning achievements and the success of online learning system implementation (Ke and Kwak, 2013). To meet learners’ real learning needs and create an effective learning environment, a growing body of literature have been conducted to examine various determinants of learner’s online satisfaction (Shen et al., 2013; Hew et al., 2020; Jiang et al., 2021).

Muilenburg and Berge (2005) identified eight barriers that prevent students from satisfactory online education: administrative and technical issues, lack of academic and technical skills, interaction, motivation, time, and support for studies, and accessibility and affordably of Internet usage. Similarly, Baber (2020) performed a comparative analysis to investigate the determinants of students' learning satisfaction on undergraduate students from South Korea and India. The study discovered that the variables such as interaction in the classroom, student engagement, course structure, teacher awareness, and facilitation positively influence students' perceived learning satisfaction. Other factors, such as online support service quality, perceived ease of use and usefulness of online platform, computer self-efficacy, academic self-efficacy, prior experience, and online learning acceptance, were found to significantly impact students’ online learning satisfaction (Lee, 2010; Jan, 2015; Jiang et al., 2021).

Among the various factors that impact learners’ online learning satisfaction and academic outcome, interaction in online learning can be seen as the key component, and its importance and effectiveness have been also emphasized by the theory of transactional distance (Moore, 1993; Benson and Samarawickrema, 2009). Even though previous studies have confirmed the positive impact of interaction on online learning satisfaction, the mechanism behind this relationship has not been well addressed in the literature. Palmer and Holt (2009) stated that the ability and the confidence to learn from online courses and connect and engage with others were the main reasons in explaining online learners' satisfaction. In this regard, this study argues that students’ academic self-efficacy and engagement in online classes may explain the relationship between interaction and online learning satisfaction.

#### Interaction

According to Moore and Kearsley (1996), interaction should be highlighted and examined in all forms of education, either face-to-face or online. It is a process that allows learners to seek new information and form connections with instructors, other learners, and content in their learning activities (Moore, 1989). It has been identified that learning activities are a significant element that critically determines the learners’ learning outcomes (Baber, 2021). A cross-country study conducted by Baber (2020) during the COVID-19 pandemic revealed interaction as the most significant factor in examining students’ online learning satisfaction and learning outcomes. It is notable that interactions in online learning have been underachieved due to technological constraints (Downing et al., 2007), and literature on distance education has largely neglected the significance of interaction (Bernard et al., 2009). Bernard et al. (2009) added that interaction has not been explicitly explained or highlighted in the study of distance education, and it is a much-needed component of online learning. Nevertheless, the study conducted by Bali and Liu (2018) has shown that in face-to-face classes, there is a higher degree of interaction and satisfaction than in online courses. Interaction can be categorized into three dimensions: interaction with instructors, interaction with peers, and interaction with content (Moore, 1989). Jung et al. (2002) found that consistent interaction with instructors accounting for 60% of students’ online satisfaction, especially in the early stages of a course. This is due to the reason that in an online learning environment, instructors are expected to offer advice, direction, and assistance to each learner based on their individual needs, to administer formal and informal evaluations, to ensure that learners are making progress, to inspire learners, and to assist learners in putting what they have learned into effect (Moore, 1989; Anderson et al., 2001). In addition, Kurucay and Inan (2017) stated that the interaction between learner-learner is also important for both student satisfaction and student academic achievement in online learning, which allows students to socialize, exchange, and discuss ideas and participate in group activities. Moreover, social interactivity with other students fosters great student satisfaction with a course (Skinner et al., 2008). In the same vein, interaction with content has been identified to be closely related to the course content quality, which in turn affects student satisfaction (Kim and Kim, 2021). The better the content quality is, the more motivated and satisfied learners are (Knowles et al., 2020). On the contrary, a few studies found that learner-learner or learner-instructor interactions have no effect on learners’ satisfaction on different Massive Open Online Courses in the United States (Kuo et al., 2014; Gameel, 2017). Thus, this study synthesizes these three components to construct interaction. Hence, we hypothesize that:

*Question (Q) 1: Is there a positive effect of interaction on online learning satisfaction?*

#### Academic Self-Efficacy

Self-efficacy is a multidimensional concept that described as an individual’s confidence on his or her ability to master a task (Bandura, 1982) and is believed to be a vital component in online learning (Shen et al., 2013). Academic self-efficacy, on the other hand, as a dimension of self-efficacy, has been defined as one’s capacity to carry out specific academic roles and attain designated performance in learning situations (Zhang, 2014). Studies have indicated that students with higher academic self-efficacy make greater progress by seeking difficult tasks and adopting effective strategies to solve those tasks (Walker et al., 2006). Specifically, those with high academic self-efficacy tend to be more academic and mastery-oriented and are devoting a greater amount of time to complete their assignments (Richardson, 2007). In contrast, students with low academic self-efficacy resulting from prior failure learning experiences tend to give up easily and are less likely to be academic engaged (Mercer et al., 2011). Moreover, extensive literature has also discovered that academic self-efficacy is closely associated with favorable academic outcomes and strongly tied to change in states of learning engagement (Zhen et al., 2017). For instance, Walker et al. (2006) found that academic self-efficacy positively impacts student engagement in the learning process. Similarly, suggest that among the motivational constructs, academic self-efficacy is one of the key players in promoting students’ engagement, including behavioral engagement, cognitive engagement, and emotional engagement.

Interestingly, most literature on academic self-efficacy focuses on its beneficial effects on the learning process and performance, and fewer studies have investigated its antecedents (Zhang, 2014). According to Bandura (1977), student academic self-efficacy can be affected by a variety of personal, cognitive, and environmental stimuli, including student’s behavior or teacher behavior, that is, interaction with teachers and peers (Zhang, 2014). In this regard, Santiago and Einarson (1998) report that graduate students’ expectations of peer/faculty interaction emerge as a significant predictor of academic self-efficacy regardless of gender differences. Further, Nelson Laird (2005) and Zhang (2014) have found that students with positive quality interaction with their peers or teachers are more likely to possess higher academic self-efficacy. In Zhang (2014)‘s study, he suggested that when university students perceived their instructors as interactive and enthusiastic, they tend to be more intrinsically motivated, which consequently fuels up their academic self-efficacy. Meanwhile, college students who experience quality positive peer-to-peer interactions are apt to possess more confidence in their academic life and tend to participate in more diversified courses in the future (Nelson Laird, 2005). Considering the compelling evidence of the positive function of academic self-efficacy and its antecedent, this study proposes the following hypotheses:

*Q2: Is there a positive effect of interaction on academic self-efficacy?*

*Q3: Is there a positive effect of academic self-efficacy on student engagement?*

#### Student Engagement

Student engagement has been referred to as the input of physical and psychological energy that a student dedicates to educationally effective activities (Astin, 1984; Kuh, 2003), which is closely related to learning outcomes, such as learning satisfaction, academic achievement, and completion rates (Baron and Corbin, 2012; Gao et al., 2020) in all modes of education (Fisher et al., 2018). According to prior research (Fredricks et al., 2016; Maroco et al., 2016), student engagement is a multidimensional construct that includes three basic substructures: behavioral, emotional, and cognitive engagement. Specifically, behavioral engagement is related to students’ behaviors, such as attending classes and participating in learning activities following the social and institutional rules (Sinval et al., 2021). Emotional engagement is referred to as the students’ positive and negative emotional responses to the learning process and class activities (Manwaring et al., 2017). Furthermore, cognitive engagement is defined as students’ learning efforts, such as learning strategies or approaches and academic self-regulation (Manwaring et al., 2017; Gao et al., 2020). In this vein, Janosz (2012) suggested that all three dimensions of students’ engagement are interdependent as students need to engage both physically (behavioral) and psychologically (emotional and cognitive) to acquire new skills and knowledge in the learning process. If students fail to engage either way in the learning process, they will be inclined to experience a low level of learning satisfaction (Sun and Rueda, 2012; Gao et al., 2020). In contrast, students who are more engaged in learning activities are more likely to spend extra time on the learning process, participate more, and develop mechanisms to assist them in the learning process and achievement (Klem and Connell, 2004; Sinval et al., 2021), which eventually led to higher learning satisfaction. This is consistent with the findings of Kim and Kim (2021) and Cheng and Chau (2016)‘s study that student engagement has a significant positive effect on students’ satisfaction. The explainable can be that most undergraduate students who were satisfied with online learning believed that active student engagement was an effective way to boost learning.

Indeed, student engagement is crucial for online pedagogy because well-designed online courses revolve around the learners (McCombs, 2015). Some studies argue that enhancing student engagement in online learning is difficult due to the overall insufficient mastery of technology and self-discipline (Oliver and Herrington, 2003). Nevertheless, Mount et al. (2009) suggest that student engagement can be best achieved by the interaction among peers and instructors. Meanwhile, some studies further discovered that student engagement mediates the impact of student interaction on students satisfaction (Jelas et al., 2016). However, contrary to prior findings, Gray and DiLoreto (2016) have argued that student engagement only mediates the effect of instructor interaction on students’ satisfaction. This medication has not been found between peer interactions and student satisfaction. This may be due to that the peer-to-peer interaction had often been identified as a poor predictor of students’ satisfaction (Kuo et al., 2014). On the other hand, academic self-efficacy has been used to predict students’ satisfaction in the online learning context (Shen et al., 2013) or as a mediator to explain the relationships between academic achievement and other factors (Hejazi et al., 2009; Shams et al., 2011). Few studies have examined its mediation effect on online learning satisfaction except on job satisfaction (Peng and Mao, 2015; Yıldız and Şimşek, 2016). Therefore, to conclude the above discussion, also with relatively limited studies on investigating the mechanism behind the relationship between interaction and online learning satisfaction and to conclude above discussions, this study predicts that:

*Q4: Is there is a positive effect of student engagement on online learning satisfaction?*

*Q5: Do academic self-efficacy and student engagement serially mediate the positive relationship between interaction and online learning satisfaction?*

## Materials and Methods

### Study Design

This study employed a cross-sectional, questionnaire-based research design to investigate the relationship between interaction and online learning satisfaction as well as the serial mediating role of academic self-efficacy and student engagement in the relationship between interaction and online learning satisfaction among Chinese university students.

### Participants

An online survey was conducted among Chinese university students from December 2020 to January 2021 using Sojump, an online questionnaire platform. The online survey link, with a brief description of the objective of the study, was shared through the Chinese social media app WeChat. Participants could respond directly from their smartphone, tablet, or laptop. The inclusion criteria for respondents were as follows: (1) Chinese university-level students who had the experience of attending online classes during the COVID-19 pandemic and (2) those who willingly participated in this study. We have used a prior estimation to calculate the minimum sample size required. The prior sample size estimation was employed during the research planning state to avoid type I and type II errors (Beck, 2013; She et al., 2021). The minimum sample size of 1,454 was required in this study based on 4 latent variables, 35 observed variables, a probability level less than 0.05, a power level of 0.8, and an effect size of 0.1 (Cohen, 2013). In total, using the convenient sampling technique, a total of 1,504 university students from five provinces in China, namely, Xinjiang, Gansu, Henan, Shandong, and Hebei, have fulfilled the inclusion criteria of this study. The sample of this study consisted of 1,058 females (70.3%) and 446 males (29.7%) with a mean age of 19.89years (*SD*=1.93). Moreover, majority of the participants were undergraduate students (97.7%) and the most of the students reported having at least six online classes per week during the pandemic (61.4%). Regarding the years in university, 83.2% of them were Year 1 and Year 2 students.

### Measures

#### Online Learning Satisfaction

Students’ online learning satisfaction was measured by adopting four items developed by Lin (2005). For the purpose of this study, “course” was replaced with “online learning” in the original scale (e.g., “The online learning activities met my expectations for what I have hoped to learn”). The participants responded on a five-point Likert scale ranging from 1 (strongly disagree) to 5 (strongly agree).

#### Interaction

To measure respondents’ interaction in online learning, this study adopted a six-item scale from Chung and Chen (2020). This is the subscale of the student perceptions of an online course. It integrates the interaction between instructors and students (e.g., “*The instructor is supportive when a student had difficulties or questions*”), between students to students (e.g., “*The course foster student-to-student interaction for supporting productive learning*”), and between content to students (e.g., “*The course content provides mutual interaction to facilitate student learning*”). *The respondents were asked to* respond on a five-point Likert scale ranging from 1 (strongly disagree) to 5 (strongly agree) to each of the statements.

#### Academic Self-Efficacy

The six-item that was adopted from Hu and Schaufeli (2009) to assess student academic self-efficacy (e.g., “I can effectively solve the problems that arise in my studies.”). This scale is a sub-dimension of the *Maslach Burnout Inventory student survey* (MBI-SS). The response was scored on a 5-point Likert scale ranging from 1 (strongly disagree) to 5 (strongly agree).

#### Student Engagement

This study used university student engagement inventory developed by Maroco et al. (2016) to measure university students engagement in online learning during the pandemic. The scale consisted of 15 items with three sub-dimensions, namely, behavior engagement (e.g., “I usually do my homework on time”), emotional engagement (e.g., “I feel excited about the school work”), and cognitive engagement (e.g., “when I read a book, I question myself to make sure I understand the subject I’m reading about”). Each item was recorded on a 5-point Likert scale ranging from one (never) to five (always). Item 6 of the scale was coded reversely (“I do not feel very accomplished at this school”).

### Data Analysis

The partial least squares structural equation modeling (PLS-SEM) and SmartPLS, version 3.3.2, were used to assess the measurement model and structural model. PLS-SEM does not impose any distribution assumptions and maximized the explained variance by the developed model (Pahlevan Sharif and Nia, 2018). PLS-SEM also allows researchers to assess more complex models with several variables, indicator constructs, and structural paths (Pahlevan Sharif et al., 2021). Hair Jr et al. (2017) indicated that if the prediction is the focus of the research, then PLS-SEM is the better option in a direct comparison with covariance-based SEM. Also, PLS-SEM and the SmartPLS software can facilitate SEM solutions with practically any level of complexity in the structural model and constructs, including higher-order constructs that usually reduce the multicollinearity issues (Ringle et al., 2014). In addition, SmartPLS software offers a wide range of algorithmic and modeling options, advanced usability with user-friendly and professional support (Bido et al., 2014). Therefore, this study employed PLS-SEM and SmartPLS software. A two-step approach was used to test the measurement model PLS-SEM also allows researchers to assess complex models that include both observed and latent construct (Pahlevan Sharif et al., 2021). A two-step approach was used to test the structural model due to the presence of both lower-order (e.g., Interaction, academic self-efficacy, online learning satisfaction) and higher-order (e.g., *student engagement*) construct (Becker et al., 2012). The internal consistency among items and construct reliability was assessed using Cronbach’s alpha and composite reliability (CR). If the value of Cronbach’s alpha and CR are more than 0.7 that indicate each test item measures the same latent trait on the same scale (Pahlevan Sharif et al., 2019; She et al., 2021). Construct validity was assessed through both convergent and discriminant validity. Convergent validity is the convergence or correlation between items that are intended to measure the same variable (Trockel et al., 2018). In other words, testing of convergent validity assumes that the items under the construct are related to the same concept. To assess and establish the convergent validity, the average variance extracted (AVE) of each construct should be greater than 0.5 and less than its CR (Sharif et al., 2019). Discriminant validity is the divergence of lack of correlation between variables that intended to assess different concepts (Trockel et al., 2018), and it is referring to the extent to which the construct is differing from one another in the research model (Henseler et al., 2015). To assess and establish the discriminant validity, the square root of each construct’s AVE should be greater than its correlation with other constructs (Fornell and Larcker, 1981). Next, the structural model was assessed. PLS algorithm was used to compute the path coefficients, and a bootstrapping approach with 2,000 subsamples was used to estimate the standard error and *value of p*. This study also used the Blindfolding procedure to obtain the Q2 value to assess the predictive accuracy of the model. All tests were two-tailed, and a *value of p* less than 0.05 is considered statistically significant.

## Results

The results of the measurement model assessment are shown in Table 1. Two items (item 6 and 8) from Emotional engagement were removed due to weak factor loadings. All the remaining items’ factor loadings were significant and greater than 0.7 for both lower-order and higher-order constructs. The internal consistency and construct reliability for all constructs were good, as evidenced by Cronbach’s alpha (ranged from 0.867 to 0.950) and CR (ranged from 0.919 to 0.959) of all constructs of greater than 0.7. In terms of convergent validity, AVE for all constructs was greater than 0.5 (ranged from 0.627 to 0.913), and each construct’s AVE was less than its respective CR, indicating good convergent validity. As shown in Table 2, the discriminant validity was also established as the square root of each construct’s AVE was greater than its correlation with other constructs.

**Table 1:** Results of the measurement model assessment.

Construct	Factor loading	Cronbach's alpha	CR	AVE
**First-order construct**				
**Interaction**				
Item 1	0.841	0.939	0.952	0.767
Item 2	0.889			
Item 3	0.916			
Item 4	0.888			
Item 5	0.864			
Item 6	0.855			
**Academic Self-Efficacy**				
Item 1	0.815	0.949	0.959	0.798
Item 2	0.743			
Item 3	0.771			
Item 4	0.886			
Item 5	0.847			
Item 6	0.878			
**Behavioral Engagement**				
Item 1	0.799	0.904	0.929	0.722
Item 2	0.837			
Item 3	0.852			
Item 4	0.875			
Item 5	0.884			
**Emotional Engagement**				
Item 7	0.879	0.867	0.919	0.791
Item 9	0.911			
Item 10	0.877			
**Cognitive Engagement**				
Item 11	0.865	0.919	0.940	0.757
Item 12	0.812			
Item 13	0.877			
Item 14	0.906			
Item 15	0.888			
**Online Learning**				
**Satisfaction**				
Item 1	0.881	0.923	0.945	0.811
Item 2	0.884			
Item 3	0.925			
Item 4	0.913			
**Second-order construct**				
**Student Engagement**				
Behavioral Engagement	0.897	0.950	0.956	0.627
Emotional Engagement	0.905			
Cognitive Engagement	0.935			

**Table 2:** Discriminant validity assessment using the Fornell-Larcker criterion.

	**Construct**	(1)	(2)	(3)	(4)	(5)	(6)	(7)
**Fornell-Larcker Criterion**	**First-order construct**							
	(1) Interaction	0.876						
	(2) Academic Self-Efficacy	0.792	0.893					
	(3) Behavioral Engagement	0.646	0.621	0.850				
	(4) Emotional Engagement	0.685	0.721	0.724	0.889			
	(5) Cognitive Engagement	0.711	0.742	0.720	0.812	0.870		
	(6) Online Learning Satisfaction	0.575	0.442	0.419	0.468	0.466	0.901	
	**Second-order construct**							
	(7) Student Engagement	0.745	0.759	–	–	–	0.493	0.792

**Figure 1:**
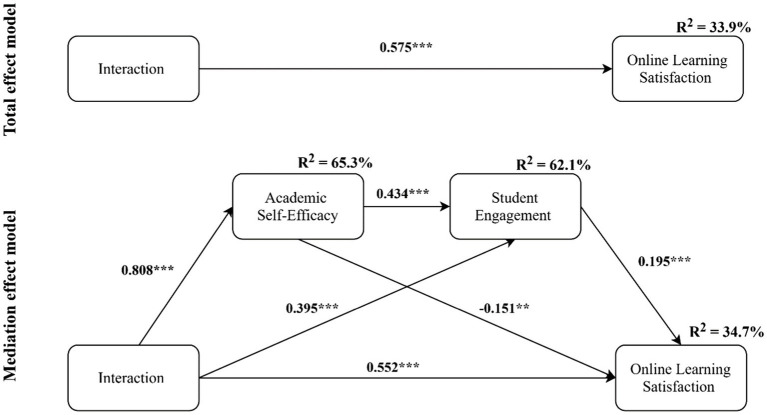
The results of the structural model. ^***^ *p*<0.001, ^**^ *p*<0.01; model controls age, gender, class per week, and years in university.

Table 3 reports the results of the structural model assessment after controlling the effect of age, gender, class per week, and years in university. Results of total effect model showed a positive relationship between interaction and online learning satisfaction (*β*=0.549, *t*-value=24.813, *p*<0.001), providing support Q1. The total effect explained 32.3% of the variance. Moreover, the results showed that the relationship between interaction and academic self-efficacy (*β*=0.792, *t*-value=56.672, *p*<0.001), academic self-efficacy and student engagement (*β*=0.759, *t*-value=49.206, *p*<0.001), and student engagement and online learning satisfaction (*β*=0.198, *t*-value=5.718, *p*<0.001) was positive and statistically significant that supported Q2, Q3, and Q4 respective. In addition, the results of the mediation model showed that there is a serial mediation of academic self-efficacy and student engagement in the relationship between interaction and online learning satisfaction (*β*=0.119, *t*-value=5.681, *p*<0.001), which support Q5. The significant relationship between interaction and online learning satisfaction (*β*=0.430, *t*-value=12.094, *p*<0.001), in the mediation model, indicated that the mediation was partial. The mediation model explained 34.6% of the variance of the online learning satisfaction, 57.6% of the variance of student engagement, and 62.7% of the variance of the academic self-efficacy (see Figure 1). The Q2 value of online learning satisfaction (31.1%), student engagement (35.8%), and academic self-efficacy (49.6%) in the mediation model showed good predictive accuracy.

**Table 3:** Structural model assessment.

**Paths**	**Standardized path coefficients**	***t*-value**	**95% confidence level (lower bound, upper bound)**
*Total effect model*			
Interaction → Online Learning satisfaction	0.575^***^	29.781	(0.535, 0.612)
*Serial mediation model*			
Interaction → Academic self-efficacy	0.808^***^	62.557	(0.783, 0.834)
Academic self-efficacy → Student engagement	0.434^***^	11.455	(0.356, 0.506)
Student engagement → Online Learning satisfaction	0.195^***^	5.342	(0.123, 0.265)
Interaction → Online Learning satisfaction	0.552^***^	12.228	(0.463, 0.640)
Interaction → Student engagement	0.395^***^	11.002	(0.325, 0.466)
Academic self-efficacy → Online Learning satisfaction	−0.151^**^	3.359	(−0.239, −0.063)
Interaction → Academic self-efficacy → Student engagement	0.351^***^	11.253	(0.289, 0.412)
Academic self-efficacy → Student engagement → Online Learning satisfaction	0.085^***^	4.950	(0.052, 0.119)
Interaction → Academic self-efficacy → Student engagement → Online Learning satisfaction	0.068^***^	4.927	(0.042, 0.097)

Control variables: age, gender, class per week, and years in university.

** *p*<0.001 and

*** *p*<0.01.

## Discussion

This study attempted to examine the relationship between interaction and online learning satisfaction and investigate the serial mediation role of academic self-efficacy and student engagement in this relationship in a sample of university students in China during the COVID-19 pandemic.

The results showed a positive relationship between interaction and online learning satisfaction (Q1). The findings indicated that Chinese students who interact more often during online learning showed higher levels of learning satisfaction. The results are consistent with prior research conducted in face-to-face learning that interaction enhances student learning involvement and develops the sense of belonging in the learning process for students (Scagnoli, 2001), which positively affects students’ learning satisfaction (Jung et al., 2002; Baber, 2021), regardless of whether it is online or face-to-face (Moore and Kearsley, 1996). Additionally, in an online environment, students leverage the technology to interact without the limitation of time, place, and space to gain knowledge and skills (Kaymak and Horzum, 2013). Furthermore, various interactivities can be crucial for students to improve their learning satisfaction and learning outcomes in an online learning environment. Strauß and Rummel (2020) stated that one of the reasons for this positive relationship in university students is that interacting in online learning fosters social presence, which can be seen as students’ perception of having psychical contact with “real” people (Aragon, 2003). Then, social presence, in turn, leads to satisfaction in online learning (Kim et al., 2011).

The results also provided evidence for the positive relationship between interaction and academic self-efficacy (Q2), hence supporting the previous studies which indicated that students with more experience in interaction with their peers, instructors, and content are more likely to have a higher level of academic self-efficacy (Nelson Laird, 2005; Zhang, 2014). A study showed that student fosters academic self-efficacy by observing and interacting with others (Gebauer et al., 2020), for example, interaction with peers to academic achievement can alter a student’s academic self-efficacy by suggesting that he or she can achieve the same results (Gebauer et al., 2020). Similarly, interaction with peers helps students create opportunities to access various academic activities to experience resources and enhance their academic self-efficacy (Schunk and Mullen, 2012). Moreover, the instructor can also enhance students’ academic self-efficacy by providing guidance and persuasive support as he/she usually works as a role model to guide and steer students’ successful mastery learning experiences (Miller and Brickman, 2004; McMahon and Wernsman, 2009). It is noted that students tend to develop their cognitive ability and perspectives through interaction with course content (Moore, 1989). The same interaction helps them perform internal didactic communication with themselves when they gain information and knowledge from course materials, enabling them to improve their confidence and ability of the discipline knowledge (Goh et al., 2019).

Besides, the positive association between academic self-efficacy and student engagement was confirmed by this study (Q3), which is in line with past studies indicating that academic self-efficacy is the key motivational construct in promoting students’ behavioral, cognitive, and emotional engagement (Linnenbrink and Pintrich, 2003; Walker et al., 2006). Students with a higher level of academic self-efficacy are more likely to take challenges and be persistent in facing multiple academic problems (Liu et al., 2018), which urges students to engage more in academic activities. It is also believed that students with competency beliefs tend to develop an intrinsic interest in learning (Ryan and Patrick, 2001), which enables them to use effective and complex learning strategies to engage and involve more in learning activities (Putwain et al., 2013). Zhen et al. (2017) stated that academic self-efficacy functions as a motivational force to motivate students to use more learning strategies and improve their cognitive competency to deal with learning challenges. Thus, students with a higher level of academic self-efficacy showed higher engagement in learning activities to attain specific academic goals in the online learning environment.

In addition, this study established that student engagement produces positive effects on online learning satisfaction (Q4). This implies that students who are more engaged with their studies are more likely to be satisfied with online learning. The findings also provide further evidence for Kim and Kim (2021) that student engagement is a key factor in enhancing students’ desirable learning outcomes, positively associated with student online learning satisfaction. In accordance with the previous studies, those students who are engaged in the learning process tend to invest more during their learning, participate more in learning activities, and tend to develop mechanisms to assist them in achieving their academic goals (Klem and Connell, 2004) and leading to more satisfaction in both face-to-face and online context learning (Coetzee and Oosthuizen, 2012; El-Sayad et al., 2021). Finally, this study confirmed the partial mediation role of academic self-efficacy and student engagement in the relationship between interaction and online learning satisfaction (Q5). The findings explained the mechanism behind this relationship and implied that academic self-efficacy and student engagement partially explained why interaction positively affects online learning satisfaction. That is to say, university students who interact more are likely to foster their academic self-efficacy (Gebauer et al., 2020). Subsequently, with higher academic self-efficacy, they believe that they have sufficient ability to perform online tasks and are more engaged with their learning (El-Sayad et al., 2021), which in turn contribute to their satisfaction in online learning (Kim and Kim, 2021).

This study also gives rise to several important implications for better understanding students’ satisfaction in the online learning context during the COVID-19 pandemic. Theoretically, this study is among the first to provide empirical evidence for serial mediating roles of academic self-efficacy and student engagement in the correlation between interaction and online learning satisfaction. In this vein, the results of the study improve our understanding of the mechanism behind the relationship between interaction and online learning satisfaction. Second, in response to a call to Ekwunife-Orakwue and Teng (2014)‘s study to identify the factors from the theory of transactional distance to predict a causal pathway for the mechanism of occurrence. This study also constitutes a novel research basis for future studies aiming at capturing a comprehensive picture of online learning satisfaction. As we learned from the study, online learning requires student interactions to boost student engagement and fuel students’ academic self-efficacy to improve their learning satisfaction online.

Practically, educators or practitioners are recommended to centralize learning interactions as a core to plan, design, and deliver online learning to create a sense of community and an online environment that emphasis the students’ own contribution to the learning process. Instructors as facilitators should be acknowledged that interactions in the learning process do not only help students learn influences students’ satisfaction but also help students build their confidence in online academic life. Meanwhile, as there is a lack of a recognized system for instructional quality measurement in the online learning context in general (Margaryan et al., 2015), the findings of the study will also be able to provide some insights for policymakers or higher education institutions to rely on to improve the current e-Learning systems across the global. In particular, the study demonstrates the process of interactions translate to online learning satisfaction through academic self-efficacy and student engagement. Thus, an e-Learning system should be designed to maximize students’ autonomy and involvement in the learning process and emphasize it as an ultimate goal of learning achievement. In this vein, students gain content knowledge, improve their creativity in completing tasks, create a sense of responsibility for their learning, and eventually benefit their future job performances.

This study, however, is not without limitations. The study sample was based on university students from five provinces of China that did not represent the whole population of Chinese university students, thus limiting the generalizability of the findings. Future studies are suggested to obtain more samples that are representative. Moreover, the use of self-report measures of the instrument may be subjected to exaggeration and lead to social desirability bias. Third, the use of a cross-sectional research design could not effectively indicate causal inferences. Thus, future research may adopt longitudinal or experimental design to provide more supporting evidence about the observed relationships and their underlying mechanisms. Also, future studies are suggested to test our model in different contexts such as blended learning environments or other online leaning related domains. Future studies testing the model developed by this study may also take into account the role of technology in students’ online satisfaction. Lastly, there is no clear explanation or reason behind the scenes in the study, and the qualitative study is much needed to delve in and gain a deeper understanding of the relationship between online interaction and online learning satisfaction from students’ perspectives.

## Data Availability Statement

The data that support the findings of this study are available from the corresponding author upon reasonable request.

## Ethics Statement

The studies involving human participants were reviewed and approved by Mazandaran University of Medical Sciences Research Ethics Committee. The patients/participants provided their written informed consent to participate in this study.

## Author Contributions

PR, HS, LS, LM contributed to the study conception and design. Material preparation, data collection were performed by LS and PR. LS and HS performed the data analysis. The first draft of the manuscript was written by LM, PR, LS, AJ, and HS. All authors commented on previous versions of the manuscript. All authors read and approved the final manuscript.

## Conflict of Interest

The authors declare that the research was conducted in the absence of any commercial or financial relationships that could be construed as a potential conflict of interest.

## Publisher’s Note

All claims expressed in this article are solely those of the authors and do not necessarily represent those of their affiliated organizations, or those of the publisher, the editors and the reviewers. Any product that may be evaluated in this article, or claim that may be made by its manufacturer, is not guaranteed or endorsed by the publisher.

## References

[ref1] Adedoyin O. B. Soykan E. (2020). Covid-19 pandemic and online learning: the challenges and opportunities. Interact. Learn. Environ., 1–13. doi: 10.1080/10494820.2020.1813180

[ref2] Alqurashi E. (2019). Predicting student satisfaction and perceived learning within online learning environments. Distance Educ. 40, 133–148. doi: 10.1080/01587919.2018.1553562

[ref3] Anderson T. Liam R. Garrison D. R. Archer W. (2001). Assessing teaching presence in a computer conferencing context. J. Asynchronous Learn. Network 5, 1–17.

[ref4] Aragon S. R. (2003). Creating social presence in online environments. New Direct. Adult and Continuing Educ. 2003, 57–68. doi: 10.1002/ace.119

[ref5] Armstrong-Mensah E. Ramsey-White K. Yankey B. Self-Brown S. (2020). COVID-19 and distance learning: effects on Georgia State University School of public health students. Front. Public Health 8:576227. doi: 10.3389/fpubh.2020.576227, PMID: 33102425 PMC7546810

[ref6] Artino A. R. (2008). Motivational beliefs and perceptions of instructional quality: predicting satisfaction with online training*. J. Comput. Assist. Learn. 24, 260–270. doi: 10.1111/j.1365-2729.2007.00258.x

[ref7] Astin A. W. (1984). Student involvement: A developmental theory for higher education. J. Coll. Stud. Pers. 25, 297–308.

[ref8] Baber H. (2020). Determinants of students’ perceived learning outcome and satisfaction in online learning during the pandemic of COVID-19. J. Educ. E-Learn. Res. 7, 285–292. doi: 10.20448/journal.509.2020.73.285.292

[ref9] Baber H. (2021). Social interaction and effectiveness of the online learning – A moderating role of maintaining social distance during the pandemic COVID-19. Asian Educ. Dev. Stud. [Ahead of print]. doi: 10.1108/AEDS-09-2020-0209

[ref10] Bali S. Liu M. C. (2018). Students’ perceptions toward online learning and face-to-face learning courses. J. Phys. Conf. Ser. 1108:012094. doi: 10.1088/1742-6596/1108/1/012094

[ref11] Bandura A. (1977). Social Learning: Theory. Englewood Cliffs, NJ: Prentice Hall.

[ref12] Bandura A. (1982). Self-efficacy mechanism in human agency. Am. Psychol. 37, 122–147. doi: 10.1037/0003-066X.37.2.122

[ref13] Baron P. Corbin L. (2012). Student engagement: rhetoric and reality. High. Educ. Res. Dev. 31, 759–772. doi: 10.1080/07294360.2012.655711

[ref14] Bayham J. Fenichel E. P. (2020). Impact of school closures for COVID-19 on the US health-care workforce and net mortality: a modelling study. Lancet Public Health 5, e271–e278. doi: 10.1016/S2468-2667(20)30082-7, PMID: 32251626 PMC7270508

[ref15] Beck T. W. (2013). The importance of A priori sample size estimation in strength and conditioning research. J. Strength Cond. Res. 27, 2323–2337. doi: 10.1519/JSC.0b013e318278eea0, PMID: 23880657

[ref16] Becker J.-M. Klein K. Wetzels M. (2012). Hierarchical latent variable models in PLS-SEM: guidelines for using reflective-formative type models. Long Range Plan. 45, 359–394. doi: 10.1016/j.lrp.2012.10.001

[ref17] Benson R. Samarawickrema G. (2009). Addressing the context of e-learning: using transactional distance theory to inform design. Distance Educ. 30, 5–21. doi: 10.1080/01587910902845972

[ref18] Bernard R. M. Abrami P. C. Borokhovski E. Wade C. A. Tamim R. M. Surkes M. A.. (2009). A meta-analysis of three types of interaction treatments in distance education. Rev. Educ. Res. 79, 1243–1289. doi: 10.3102/0034654309333844

[ref20] Bong M. Skaalvik E. M. (2003). Academic Self-concept and Self-efficacy: how different are they really? Educ. Psychol. Rev. 15, 1–40. doi: 10.1023/A:1021302408382

[ref21] Cheng G. Chau J. (2016). Exploring the relationships between learning styles, online participation, learning achievement and course satisfaction: An empirical study of a blended learning course. Br. J. Educ. Technol. 47, 257–278. doi: 10.1111/bjet.12243

[ref22] Chung J. Chen H.-C. (2020). Development and psychometric properties of student perceptions of an online course (SPOC) in an RN-to-BSN program. Nurse Educ. Today 85:104303. doi: 10.1016/j.nedt.2019.104303, PMID: 31785574

[ref23] Cidral W. A. Oliveira T. Di Felice M. Aparicio M. (2018). E-learning success determinants: Brazilian empirical study. Comput. Educ. 122, 273–290. doi: 10.1016/j.compedu.2017.12.001

[ref24] Coetzee M. Oosthuizen R. M. (2012). Students' sense of coherence, study engagement and Self-efficacy in relation to their study and employability satisfaction. J. Psychol. Afr. 22, 315–322. doi: 10.1080/14330237.2012.10820536

[ref25] Cohen J. (2013). Statistical Power Analysis for the Behavioral Sciences. New York: Academic press.

[ref26] Coman C. Țîru L. G. Meseșan-Schmitz L. Stanciu C. Bularca M. C. (2020). Online teaching and learning in higher education during the coronavirus pandemic: students’ perspective. Sustainability 12:10367. doi: 10.3390/su122410367

[ref27] Downing K. J. Lam T. Kwong T. Downing W. Chan S. (2007). Creating interaction in online learning: a case study. ALT-J 15, 201–215. doi: 10.1080/09687760701673592

[ref28] Dziuban C. Moskal P. Thompson J. Kramer L. DeCantis G. Hermsdorfer A. (2015). Student satisfaction with online learning: is it a psychological contract? Online Learn. 19:n2.

[ref29] Ekwunife-Orakwue K. C. V. Teng T.-L. (2014). The impact of transactional distance dialogic interactions on student learning outcomes in online and blended environments. Comput. Educ. 78, 414–427. doi: 10.1016/j.compedu.2014.06.011

[ref30] El-Sayad G. Md Saad N. H. Thurasamy R. (2021). How higher education students in Egypt perceived online learning engagement and satisfaction during the COVID-19 pandemic. J. Computer. Educ., 1–24. doi: 10.1007/s40692-021-00191-y

[ref31] Enkin E. Mejías-Bikandi E. (2017). The effectiveness of online teaching in an advanced Spanish language course. Int. J. Appl. Linguist. 27, 176–197. doi: 10.1111/ijal.12112

[ref32] Ferla J. Valcke M. Cai Y. (2009). Academic self-efficacy and academic self-concept: reconsidering structural relationships. Learn. Individ. Differ. 19, 499–505. doi: 10.1016/j.lindif.2009.05.004

[ref33] Fisher R. Perényi Á. Birdthistle N. (2018). The positive relationship between flipped and blended learning and student engagement, performance and satisfaction. Active Learn. High. Educ. 22, 97–113. doi: 10.1177/1469787418801702

[ref34] Fornell C. Larcker D. F. (1981). Evaluating structural equation models with unobservable variables and measurement error. J. Mark. Res. 18, 39–50. doi: 10.1177/002224378101800104

[ref36] Fredricks J. A. Filsecker M. Lawson M. A. (2016). Student engagement, context, and adjustment: addressing definitional, measurement, and methodological issues. Learn. Instr. 43, 1–4. doi: 10.1016/j.learninstruc.2016.02.002

[ref37] Gameel B. G. (2017). Learner satisfaction with massive open online courses. Am. J. Dist. Educ. 31, 98–111. doi: 10.1080/08923647.2017.1300462

[ref38] Gao B. W. Jiang J. Tang Y. (2020). The effect of blended learning platform and engagement on students’ satisfaction— the case from the tourism management teaching. J. Hosp. Leis. Sport Tour. Educ. 27:100272. doi: 10.1016/j.jhlste.2020.100272

[ref39] Gebauer M. M. McElvany N. Bos W. Köller O. Schöber C. (2020). Determinants of academic self-efficacy in different socialization contexts: investigating the relationship between students’ academic self-efficacy and its sources in different contexts. Soc. Psychol. Educ. 23, 339–358. doi: 10.1007/s11218-019-09535-0

[ref40] Goh C. F. Tan O. K. Rasli A. Choi S. L. (2019). Engagement in peer review, learner-content interaction and learning outcomes. Int. J. Info. Learn. Technol. 36, 423–433. doi: 10.1108/IJILT-04-2018-0038

[ref41] Gopal R. Singh V. Aggarwal A. (2021). Impact of online classes on the satisfaction and performance of students during the pandemic period of COVID 19. Educ. Inf. Technol., 1–25. doi: 10.1007/s10639-021-10523-1, PMID: 33903795 PMC8059127

[ref42] Gray J. A. DiLoreto M. (2016). The effects of student engagement, student satisfaction, and perceived learning in online learning environments. Int. J. Educ. Leadership Prepar. 11:n1

[ref43] Hair J. F. Jr. Matthews L. M. Matthews R. L. Sarstedt M. (2017). PLS-SEM or CB-SEM: updated guidelines on which method to use. Int. J. Multivariate Data Analy. 1, 107–123. doi: 10.1504/IJMDA.2017.10008574

[ref44] Hassan S. U. Algahtani F. D. Zrieq R. Aldhmadi B. K. Atta A. Obeidat R. M.. (2021). Academic Self-perception and course satisfaction among university students taking virtual classes during the COVID-19 pandemic in the kingdom of Saudi-Arabia (KSA). Educ. Sci. 11:134. doi: 10.3390/educsci11030134

[ref45] Hejazi E. Shahraray M. Farsinejad M. Asgary A. (2009). Identity styles and academic achievement: mediating role of academic self-efficacy. Soc. Psychol. Educ. 12, 123–135. doi: 10.1007/s11218-008-9067-x

[ref46] Henseler J. Ringle C. M. Sarstedt M. (2015). A new criterion for assessing discriminant validity in variance-based structural equation modeling. J. Acad. Mark. Sci. 43, 115–135. doi: 10.1007/s11747-014-0403-8

[ref47] Hew K. F. Hu X. Qiao C. Tang Y. (2020). What predicts student satisfaction with MOOCs: A gradient boosting trees supervised machine learning and sentiment analysis approach. Comput. Educ. 145:103724. doi: 10.1016/j.compedu.2019.103724

[ref48] Hu Q. Schaufeli W. B. (2009). The factorial validity of the Maslach burnout inventory–student survey in China. Psychol. Rep. 105, 394–408. doi: 10.2466/PR0.105.2.394-408, PMID: 19928601

[ref49] Jan S. K. (2015). The relationships Between academic Self-efficacy, computer Self-efficacy, prior experience, and satisfaction With online learning. Am. J. Dist. Educ. 29, 30–40. doi: 10.1080/08923647.2015.994366

[ref50] Jan A. (2020). A phenomenological study of synchronous teaching during COVID-19: A case of an international school in Malaysia. Soc. Sci. Human. open 2:100084. doi: 10.1016/j.ssaho.2020.100084, PMID: 34173504 PMC7657005

[ref51] Janosz M. (2012). “Part IV commentary: outcomes of engagement and engagement as an outcome: Some consensus, divergences, and unanswered questions” in Handbook of Research on Student Engagement. eds. Christenson S. L. Reschly A. L. Wylie C. (Boston, MA: Springer US), 695–703.

[ref52] Jelas Z. M. Azman N. Zulnaidi H. Ahmad N. A. (2016). Learning support and academic achievement among Malaysian adolescents: the mediating role of student engagement. Learn. Environ. Res. 19, 221–240. doi: 10.1007/s10984-015-9202-5

[ref53] Jiang H. Islam A. Y. M. A. Gu X. Spector J. M. (2021). Online learning satisfaction in higher education during the COVID-19 pandemic: A regional comparison between eastern and Western Chinese universities. Educ. Inf. Technol., 1–23. doi: 10.1007/s10639-021-10519-x, PMID: 33814959 PMC8010491

[ref54] Jung I. Choi S. Lim C. Leem J. (2002). Effects of different types of interaction on learning achievement, satisfaction and participation in web-based instruction. Innov. Educ. Teach. Int. 39, 153–162. doi: 10.1080/14703290252934603

[ref55] Jung Y. Lee J. (2018). Learning engagement and persistence in massive open online courses (MOOCS). Comput. Educ. 122, 9–22. doi: 10.1016/j.compedu.2018.02.013

[ref56] Kaymak Z. D. Horzum M. B. (2013). Relationship between online learning readiness and structure and interaction of online learning students. Educ. Sci. Theory Practice 13, 1792–1797.

[ref57] Ke F. Kwak D. (2013). Online learning across ethnicity and age: A study on learning interaction participation, perception, and learning satisfaction. Comput. Educ. 61, 43–51. doi: 10.1016/j.compedu.2012.09.003

[ref58] Kim S. Kim D.-J. (2021). Structural relationship of key factors for student satisfaction and achievement in asynchronous online learning. Sustainability 13:6734. doi: 10.3390/su13126734

[ref59] Kim J. Kwon Y. Cho D. (2011). Investigating factors that influence social presence and learning outcomes in distance higher education. Comput. Educ. 57, 1512–1520. doi: 10.1016/j.compedu.2011.02.005

[ref60] Klem A. M. Connell J. P. (2004). Relationships matter: linking teacher support to student engagement and achievement. J. Sch. Health 74, 262–273. doi: 10.1111/j.1746-1561.2004.tb08283.x 15493703

[ref61] Knowles M. S. Holton Iii E. F. Swanson R. A. (2020). The Adult Learner: The Definitive Classic in Adult Education and Human Resource Development. 9th *Edn*. New York: Routledge.

[ref62] Kuh G. D. (2003). What We're learning About student engagement From NSSE: benchmarks for effective educational practices. Change Magazine High. Learn. 35, 24–32. doi: 10.1080/00091380309604090

[ref63] Kumar P. Saxena C. Baber H. (2021). Learner-content interaction in e-learning- the moderating role of perceived harm of COVID-19 in assessing the satisfaction of learners. Smart Learn. Environ. 8, 1–15. doi: 10.1186/s40561-021-00149-8 PMC8050475 40477398

[ref64] Kuo Y.-C. Walker A. E. Schroder K. E. E. Belland B. R. (2014). Interaction, internet self-efficacy, and self-regulated learning as predictors of student satisfaction in online education courses. Internet High. Educ. 20, 35–50. doi: 10.1016/j.iheduc.2013.10.001

[ref65] Kurucay M. Inan F. A. (2017). Examining the effects of learner-learner interactions on satisfaction and learning in an online undergraduate course. Comput. Educ. 115, 20–37. doi: 10.1016/j.compedu.2017.06.010

[ref66] Lee J.-W. (2010). Online support service quality, online learning acceptance, and student satisfaction. Internet High. Educ. 13, 277–283. doi: 10.1016/j.iheduc.2010.08.002

[ref67] Lin Y.-M. (2005). Understanding Students' Technology Appropriation and Learning Perceptions in Online Learning Environments. Columbia: University of Missouri. Doctoral dissertation.

[ref68] Linnenbrink E. A. Pintrich P. R. (2003). The role of self-efficacy beliefs INSTUDENT engagement and learning INTHECLASSROOM. Read. Writ. Q. 19, 119–137. doi: 10.1080/10573560308223

[ref69] Liu R.-D. Zhen R. Ding Y. Liu Y. Wang J. Jiang R.. (2018). Teacher support and math engagement: roles of academic self-efficacy and positive emotions. Educ. Psychol. 38, 3–16. doi: 10.1080/01443410.2017.1359238

[ref70] Manwaring K. C. Larsen R. Graham C. R. Henrie C. R. Halverson L. R. (2017). Investigating student engagement in blended learning settings using experience sampling and structural equation modeling. Internet High. Educ. 35, 21–33. doi: 10.1016/j.iheduc.2017.06.002

[ref71] Margaryan A. Bianco M. Littlejohn A. (2015). Instructional quality of massive open online courses (MOOCs). Comput. Educ. 80, 77–83. doi: 10.1016/j.compedu.2014.08.005

[ref72] Maroco J. Maroco A. L. Campos J. A. D. B. Fredricks J. A. (2016). University student’s engagement: development of the university student engagement inventory (USEI). Psicologia: Reflexão e Crítica 29:21. doi: 10.1186/s41155-016-0042-8

[ref73] Martin F. Wang C. Sadaf A. (2018). Student perception of helpfulness of facilitation strategies that enhance instructor presence, connectedness, engagement and learning in online courses. Internet High. Educ. 37, 52–65. doi: 10.1016/j.iheduc.2018.01.003

[ref74] McCombs B. (2015). Learner-Centered online instruction. New Dir. Teach. Learn. 2015, 57–71. doi: 10.1002/tl.20163

[ref75] McMahon S. D. Wernsman J. (2009). The relation of classroom environment and school belonging to academic Self-efficacy among urban fourth- and fifth-grade students. Elem. Sch. J. 109, 267–281. doi: 10.1086/592307

[ref76] Mercer S. H. Nellis L. M. Martínez R. S. Kirk M. (2011). Supporting the students most in need: academic self-efficacy and perceived teacher support in relation to within-year academic growth. J. Sch. Psychol. 49, 323–338. doi: 10.1016/j.jsp.2011.03.006, PMID: 21640247

[ref77] Miller R. B. Brickman S. J. (2004). A model of future-oriented motivation and Self-regulation. Educ. Psychol. Rev. 16, 9–33. doi: 10.1023/B:EDPR.0000012343.96370.39

[ref78] Moore M. G. (1989). Editorial: three types of interaction. Am. J. Dist. Educ. 3, 1–7. doi: 10.1080/08923648909526659

[ref79] Moore M. G. (1993). “Theory of transactional distance,” in Theoretical Principles of Distance Education. ed. Keegan D. (New York: Routledge), 84–103.

[ref80] Moore M. G. Kearsley G. G. (1996). Distance Education: A System View. Wadsworth: Wadsworth Publishing Company.

[ref81] Moore M. G. Kearsley G. (2011). Distance Education: A Systems View of Online Learning. 3rd *Edn*. Wadsworth: Cengage Learning.

[ref82] Mount N. J. Chambers C. Weaver D. Priestnall G. (2009). Learner immersion engagement in the 3D virtual world: principles emerging from the DELVE project. Innovat. Teach. Learn. Info. Comput. Sci. 8, 40–55. doi: 10.11120/ital.2009.08030040

[ref83] Muilenburg L. Y. Berge Z. L. (2005). Student barriers to online learning: A factor analytic study. Distance Educ. 26, 29–48. doi: 10.1080/01587910500081269

[ref84] Nelson Laird T. F. (2005). College students’ experiences with diversity and their effects on academic Self-confidence, social agency, and disposition toward critical thinking. Res. High. Educ. 46, 365–387. doi: 10.1007/s11162-005-2966-1

[ref85] OECD. (2020). Strengthening online learning when schools are closed: The role of families and teachers in supporting students during the COVID-19 crisis. Available at: https://www.oecd.org/coronavirus/policy-responses/strengthening-online-learning-when-schools-are-closed-the-role-of-families-and-teachers-in-supporting-students-during-the-covid-19-crisis-c4ecba6c/ (Accessed June 10, 2021).

[ref86] Oliver R. Herrington J. (2003). “Factors influencing quality online learning experiences,” in Quality Education @ a Distance: IFIP TC3/WG3.6 Working Conference on Quality Education @ a Distance. eds. Davies G. Stacey E. February 3–6, 2003; Geelong, Australia (Boston, MA: Springer US), 129–136.

[ref104] Pahlevan Sharif S. Mostafiz I. Guptan V. (2019). A systematic review of structural equation modelling in nursing research. Nurs. Res. 26, 28–31. doi: 10.7748/nr.2018.e1577, PMID: 30207432

[ref105] Pahlevan Sharif S. Nia H. S. (2018). Structural Equation Modeling with AMOS. Tehran: Artin Teb.

[ref87] Pahlevan Sharif S. Naghavi N. Ong Fon S. Sharif Nia H. Waheed H. (2021). Health insurance satisfaction, financial burden, locus of control and quality of life of cancer patients: a moderated mediation model. Int. J. Soc. Econ. 48, 513–530. doi: 10.1108/IJSE-10-2019-0629

[ref88] Palmer S. R. Holt D. M. (2009). Examining student satisfaction with wholly online learning. J. Comput. Assist. Learn. 25, 101–113. doi: 10.1111/j.1365-2729.2008.00294.x

[ref89] Parahoo S. K. Santally M. I. Rajabalee Y. Harvey H. L. (2016). Designing a predictive model of student satisfaction in online learning. J. Mark. High. Educ. 26, 1–19. doi: 10.1080/08841241.2015.1083511

[ref90] Peng Y. Mao C. (2015). The impact of person–job fit on job satisfaction: The mediator role of Self efficacy. Soc. Indic. Res. 121, 805–813. doi: 10.1007/s11205-014-0659-x

[ref91] Putwain D. Sander P. Larkin D. (2013). Academic self-efficacy in study-related skills and behaviours: relations with learning-related emotions and academic success. Br. J. Educ. Psychol. 83, 633–650. doi: 10.1111/j.2044-8279.2012.02084.x 24175686

[ref92] Rabin E. Kalman Y. M. Kalz M. (2019). An empirical investigation of the antecedents of learner-centered outcome measures in MOOCs. Int. J. Educ. Technol. High. Educ. 16, 1–20. doi: 10.1186/s41239-019-0144-3

[ref93] Rahmatpour P. Peyrovi H. Sharif Nia H. (2021). Development and psychometric evaluation of postgraduate nursing student academic satisfaction scale. Nur. Open 8, 1145–1156. doi: 10.1002/nop2.727, PMID: 34482656 PMC8046041

[ref94] Richardson J. T. E. (2007). Motives, attitudes and approaches to studying in distance education. High. Educ. 54, 385–416. doi: 10.1007/s10734-006-9003-y

[ref19] Ringle C. Da Silva D. Bido D. (2015). Structural equation modeling with the smartpls. Brazilian J. Market. 13, 56–73. doi: 10.5585/remark.v13i2.2717

[ref95] Robinson C. C. Hullinger H. (2008). New benchmarks in higher education: student engagement in online learning. J. Educ. Bus. 84, 101–109. doi: 10.3200/JOEB.84.2.101-109

[ref96] Ryan A. M. Patrick H. (2001). The classroom social environment and changes in adolescents’ motivation and engagement During middle school. Am. Educ. Res. J. 38, 437–460. doi: 10.3102/00028312038002437

[ref97] Sahu P. (2020). Closure of universities due to coronavirus disease 2019 (COVID-19): Impact on Education and mental health of students and academic staff. Cureus 12:e7541. doi: 10.7759/cureus.7541, PMID: 32377489 PMC7198094

[ref98] Santiago A. M. Einarson M. K. (1998). Background characteristics as predictors of academic self-confidence and academic self-efficacy among graduate science and engineering students. Res. High. Educ. 39, 163–198. doi: 10.1023/A:1018716731516

[ref99] Scagnoli N. I. (2001). Student orientations for online programs. J. Res. Technol. Educ. 34, 19–27. doi: 10.1080/15391523.2001.10782330

[ref100] Schunk D. H. Mullen C. A. (2012). “Self-efficacy as an engaged learner,” in Handbook of Research on Student Engagement. eds. Christenson S. L. Reschly A. L. Wylie C. (Boston, MA: Springer US), 219–235.

[ref101] Selvanathan M. Hussin N. A. M. Azazi N. A. N. (2020). Students learning experiences during COVID-19: work from home period in Malaysian higher learning institutions. Teach. Public Admin.:0144739420977900. doi: 10.1177/0144739420977900

[ref102] Shahzad A. Hassan R. Aremu A. Y. Hussain A. Lodhi R. N. (2021). Effects of COVID-19 in E-learning on higher education institution students: the group comparison between male and female. Qual. Quant. 55, 805–826. doi: 10.1007/s11135-020-01028-z, PMID: 32836471 PMC7402545

[ref103] Shams F. Mooghali A. R. Soleimanpour N. (2011). The mediating role of academic self-efficacy in the relationship between personality traits and mathematics performance. Procedia. Soc. Behav. Sci. 29, 1689–1692. doi: 10.1016/j.sbspro.2011.11.413

[ref106] She L. Rasiah R. Waheed H. Pahlevan Sharif S. (2021). Excessive use of social networking sites and financial well-being among young adults: the mediating role of online compulsive buying. Young Consum. 22, 272–289. doi: 10.1108/YC-11-2020-1252

[ref107] She L. Sharif S. P. Nia H. S. (2021). Psychometric evaluation of the Chinese version of the modified online compulsive buying scale among Chinese young consumers. J. Asia Pac. Bus. 22, 121–133. doi: 10.1080/10599231.2021.1905493

[ref108] Shen D. Cho M.-H. Tsai C.-L. Marra R. (2013). Unpacking online learning experiences: online learning self-efficacy and learning satisfaction. Internet High. Educ. 19, 10–17. doi: 10.1016/j.iheduc.2013.04.001

[ref109] Sinval J. Casanova J. R. Marôco J. Almeida L. S. (2021). University student engagement inventory (USEI): psychometric properties. Curr. Psychol. 40, 1608–1620. doi: 10.1007/s12144-018-0082-6

[ref110] Skinner E. Furrer C. Marchand G. Kindermann T. (2008). Engagement and disaffection in the classroom: part of a larger motivational dynamic? J. Educ. Psychol. 100, 765–781. doi: 10.1037/a0012840

[ref111] Strauß S. Rummel N. (2020). Promoting interaction in online distance education: designing, implementing and supporting collaborative learning. Inf. Learn. Sci. 121, 251–260. doi: 10.1108/ILS-04-2020-0090

[ref112] Sun J. C.-Y. Rueda R. (2012). Situational interest, computer self-efficacy and self-regulation: their impact on student engagement in distance education. Br. J. Educ. Technol. 43, 191–204. doi: 10.1111/j.1467-8535.2010.01157.x

[ref113] Topala I. Tomozii S. (2014). Learning satisfaction: validity and reliability testing for students’ learning satisfaction questionnaire (SLSQ). Procedia. Soc. Behav. Sci. 128, 380–386. doi: 10.1016/j.sbspro.2014.03.175

[ref114] Trockel M. Bohman B. Lesure E. Hamidi M. S. Welle D. Roberts L.. (2018). A brief instrument to assess Both burnout and professional Fulfillment in physicians: reliability and validity, including correlation with Self-reported medical errors, in a sample of resident and practicing physicians. Acad. Psychiatry 42, 11–24. doi: 10.1007/s40596-017-0849-3, PMID: 29196982 PMC5794850

[ref115] Veletsianos G. (2010). Contextually relevant pedagogical agents: visual appearance, stereotypes, and first impressions and their impact on learning. Comput. Educ. 55, 576–585. doi: 10.1016/j.compedu.2010.02.019

[ref116] Walker C. O. Greene B. A. Mansell R. A. (2006). Identification with academics, intrinsic/extrinsic motivation, and self-efficacy as predictors of cognitive engagement. Learn. Individ. Differ. 16, 1–12. doi: 10.1016/j.lindif.2005.06.004

[ref117] Wang G. Zhang Y. Zhao J. Zhang J. Jiang F. (2020). Mitigate the effects of home confinement on children during the COVID-19 outbreak. Lancet 395, 945–947. doi: 10.1016/S0140-6736(20)30547-X, PMID: 32145186 PMC7124694

[ref118] Wu J.-H. Tennyson R. D. Hsia T.-L. (2010). A study of student satisfaction in a blended e-learning system environment. Comput. Educ. 55, 155–164. doi: 10.1016/j.compedu.2009.12.012

[ref35] World Economic Forum (2020). The COVID-19 pandemic has changed education forever. This is how Available at: https://www.weforum.org/agenda/2020/04/coronavirus-education-global-covid19-online-digital-learning/ (Accessed June 10, 2021).

[ref119] Yıldız I. G. Şimşek Ö. F. (2016). Different pathways from transformational leadership to job satisfaction. Nonprofit Manage. Leader. 27, 59–77. doi: 10.1002/nml.21229

[ref120] Zhang Q. (2014). Assessing the effects of instructor enthusiasm on classroom engagement, learning goal orientation, and academic Self-efficacy. Commun. Teach. 28, 44–56. doi: 10.1080/17404622.2013.839047

[ref121] Zhen R. Liu R.-D. Ding Y. Wang J. Liu Y. Xu L. (2017). The mediating roles of academic self-efficacy and academic emotions in the relation between basic psychological needs satisfaction and learning engagement among Chinese adolescent students. Learn. Individ. Differ. 54, 210–216. doi: 10.1016/j.lindif.2017.01.017

